# Generalized Treatment as Prevention Plus Focused Pre-Exposure Prophylaxis Is the Key to Controlling HIV/AIDS

**DOI:** 10.3390/tropicalmed10030075

**Published:** 2025-03-12

**Authors:** Julio S. G. Montaner, Viviane D. Lima, Kate A. Salters, Junine Toy, Jeffrey B. Joy, Silvia Guillemi, Rolando Barrios

**Affiliations:** 1 British Columbia Centre for Excellence in HIV/AIDS, Vancouver, BC V6Z 1Y6, Canada; 2 Faculty of Medicine, University of British Columbia, Vancouver, BC V6T 1Z4, Canada

**Keywords:** HIV/AIDS prevention, Treatment as Prevention (TasP), Pre-Exposure Prophylaxis (PrEP), HIV control strategies, HIV treatment cascade, antiretroviral therapy (ART), HIV transmission prevention, HIV epidemiology, targeted interventions in HIV, public health and HIV/AIDS

## Abstract

Treatment as Prevention (TasP) and Pre-Exposure Prophylaxis (PrEP) are both widely recognized as essential biomedical tools to control HIV/AIDS. TasP calls for the immediate initiation of fully subsidized and supported antiretroviral therapy (ART) following HIV diagnosis. TasP effectively prevents progression to AIDS, and premature AIDS-related deaths among people living with HIV (PLWH), and simultaneously renders HIV non-transmissible, thus preventing onward HIV transmission. In addition, PrEP has proven effective against HIV transmission among high-risk individuals who are adherent to the regimen. PrEP traditionally consists of two antiretrovirals given orally as one pill daily: originally, tenofovir-DF plus emtricitabine (TDF-FTC), and later, tenofovir-AF (TAF) plus FTC (more recently, other options have become available, including long-acting parenteral formulations; however, these are still of limited availability). Over the last two decades, the province of British Columbia has rolled out TasP among all PLWH, and starting in 2018, PrEP was added as a strategy to reach individuals most at risk of acquiring HIV to further accelerate progress in addressing HIV/AIDS as a public health threat. Our “generalized TasP + focused PrEP” program proved to be synergistic (or multiplicative) as it relates to reducing the HIV effective reproduction number ($R_{e}$). TasP lowers HIV incidence by reducing the pool of individuals able to transmit HIV, which is dependent on the extent of community plasma viral load (pVL) suppression. Meanwhile, PrEP reduces the number of potential new infections among those most susceptible to acquiring HIV in the community, independent of viral load suppression among PLWH. Our results strongly support widespread implementation of the combination of “generalized TasP + focused PrEP” strategy and underscore the importance of long-term monitoring of $R_{e}$ at a programmatic level to identify opportunities for optimizing TasP and PrEP programs. This approach aligns with the United Nations goal of “Ending HIV/AIDS as a pandemic by 2030”, both in Canada and globally.

## 1. Introduction

Since 1996, modern antiretroviral therapy (ART) has markedly increased AIDS-free survival among people living with HIV (PLWH) [1,2]. Currently, newly diagnosed PLWH can realistically expect near-normal AIDS-free longevity, provided they have access to effective HIV-related healthcare and support, including, importantly, free-of-charge ART without restriction [3,4].

## 2. Treatment as Prevention

In 2006 [5] and 2009 [6], two independent Lancet reports proposed Treatment as Prevention (TasP) as a global HIV/AIDS control strategy. This built on previous reports suggesting that ART could play a significant role, as part of a comprehensive HIV control strategy [7,8]. TasP postulates that, in addition to preventing clinical progression to AIDS and premature death, ART reduces plasma viral load (pVL) to undetectable levels in biological fluids, including blood plasma and sexual fluids [5,6,7,8,9,10,11], rendering HIV non-infectious [12]. For over two decades, observational studies from diverse settings have suggested a protective effect from ART against HIV infection [13,14,15,16]. However, the efficacy of TasP was independently validated in 2011 through the HPTN052 trial results [17], and further verified by a phylogenetic study in 2016 [18]. A recent systematic review revealed a near-zero risk of sexual transmission for PLWH on ART with pVL below 1000 copies/mL [19], leading Bekker et al. [20] to conclude that “HIV is sexually untransmittable when viral load is undetectable”.

TasP gained global recognition when Michel Sidibé, Executive Director of the Joint United Nations Programme on HIV/AIDS (UNAIDS), endorsed it as a control strategy at the 2010 Treatment as Prevention Summit in Vancouver, British Columbia (BC), Canada [21]. TasP became the foundation for UNAIDS’ Treatment 2.0 strategy [22], which in turn served as the basis for the United Nations (UN) 90-90-90 by 2020 target [23] and subsequent UN 95-95-95 by 2025 target [24]. These initiatives, originally proposed by the BC Centre for Excellence in HIV/AIDS (BC-CfE), were designed with the specific aim of achieving a 90% reduction in AIDS-related deaths and HIV incidence globally by 2030 [25].

## 3. Pre-Exposure Prophylaxis

In 2010, the iPrEx trial of oral Pre-Exposure Prophylaxis (PrEP) showed a 44% reduction in HIV incidence (95% confidence interval 15–63; *p* = 0.005) among gay and bisexual men who have sex with men (gbMSM) at elevated risk of HIV infection [26]. However, a 2012 post hoc sub-study revealed that none of the HIV incident cases in the original study had adequate PrEP drug levels at the time of HIV diagnosis [27]. In July 2012, the United States Food and Drug Administration approved the use of daily emtricitabine plus tenofovir-DF (FTC-TDF) as PrEP for preventing sexually acquired HIV infection among gbMSM at elevated risk of HIV infection. More recently, on-demand PrEP has emerged as an effective alternative strategy, offering greater flexibility to individuals at risk [28,29]. In 2020, daily FTC plus tenofovir alafenamide (TAF) showed non-inferior efficacy to daily FTC-TDF for HIV prevention. While the frequency of adverse events for both oral PrEP regimens was low, the authors noted FTC-TAF had more favourable effects on bone mineral density and biomarkers of renal safety than FTC-TDF. However, this came at a substantially higher cost; therefore, in some settings, including in British Columbia, FTC-TDF remains the preferred PrEP option, with FTC-TAF reserved for individual cases where TDF is either contraindicated (i.e., renal or bone disease) or not tolerated. As a result, the outcomes presented here were obtained with over 90% of PrEP prescriptions receiving Truvada^®^. HIV-PrEP was evaluated in women within a randomized, placebo-controlled trial comparing oral TDF alone, oral FTC-TDF, or 1% tenofovir vaginal (TFV) in South Africa, Uganda, and Zimbabwe [30]. HIV-1 testing was performed monthly, and plasma TFV levels were assessed quarterly. With over 5000 participants enrolled, the study showed that none of the regimens evaluated reduced the rates of HIV infection; however, adherence to study drugs was low, with less than 30% of available plasma samples from participants showing the presence of the study drug. Thus, the precise role of PrEP among women remains to be fully elucidated, and in the interim, it is recommended this be discussed with potentially interested parties, and a way forward decided on a case-by-case basis [31].

In 2015, the World Health Organization [32], along with other international health agencies [33,34,35], endorsed the combined use of TasP and PrEP as key strategies for global control of HIV/AIDS. These initiatives were subsequently incorporated into UN Sustainable Development Goals, further reinforcing their role in the international effort to end the HIV/AIDS pandemic [36]. In 2019, the FDA approved tenofovir-AF (TAF) plus emtricitabine (FTC) for PrEP [37].

More recently, the results of a phase 3, double-blind, randomized, controlled trial of HIV prevention with either subcutaneous lenacapavir q 6 months, daily oral FTC-TDF, or daily oral FTC-TAF, among adolescent girls and young women in South Africa and Uganda were reported [38]. Participants were assigned in a 2:2:1 ratio to receive subcutaneous lenacapavir every 26 weeks, daily oral FTC-TAF, or daily oral FTC-TDF. Of note, all participants also received the alternate subcutaneous or oral placebo. Among 5338 participants who were initially HIV negative, 55 incident HIV infections were observed: 0 infections among 2134 participants receiving lenacapavir, 39 among 2136 participants on FTC-TAF (2.02/100 person-years; 95% CI, 1.44 to 2.76), and 16 among 1068 participants on FTC-TDF (1.69/100 person-years; 95% CI, 0.96 to 2.74). In contrast to adherence to lenacapavir, adherence to F/TAF and F/TDF was low. While no major safety concerns were found, injection-site reactions were common in the lenacapavir group, with four participants discontinuing therapy due to injection-site reactions. A second complementary phase 3, double-blind, randomized, active-controlled trial, was recently reported. In brief, eligible participants were randomly assigned in a 2:1 ratio to receive subcutaneous lenacapavir every 26 weeks or daily oral FTC-TDF. Among 3265 participants, incident HIV infections occurred in 2 participants in the lenacapavir group (0.10 per 100 person-years; 95% confidence interval [CI], 0.01 to 0.37) and in 9 participants in the F/TDF group (0.93 per 100 person-years; 95% CI, 0.43 to 1.77). The incidence of HIV infection in the lenacapavir group was statistically significantly lower than in the F/TDF group (incidence rate ratio, 0.11; 95% CI, 0.02 to 0.51; *p* = 0.002). Treatment discontinuations due to injection-site reactions occurred in 1.2% and 0.3% of lenacapavir- and FTC-TDF-treated individuals, respectively. No new safety concerns were reported [39]. These results, impressive as they are, will not be fully realized in the real world unless the price of lenacapavir is lowered considerably [40].

## 4. Antiretroviral Therapy and PrEP Programs in British Columbia

BC offers a unique setting with its single-payer, fully subsidized, and centralized HIV/AIDS program [41]. Importantly, the BC government covers all costs related to TasP and PrEP, including medical expenses, laboratory testing, and medications. Since the establishment of the BC-CfE in 1992, the Drug Treatment Program (DTP) has provided free access to ART for over 16,500 PLWH. Between 1992 and 1996, monotherapy and dual therapy regimens were the norm. However, these regimens were only partially effective and thus were replaced by the emerging triple therapy regimens in 1996 [1,2].

Since 1996, BC has experienced several distinct ART roll-out phases, aligning with evolving therapeutic guidelines [1,2]. In the Spring of 1996, highly active antiretroviral therapy (HAART) became available for free in BC for all persons living with HIV indicated for treatment. In 2009, supported by the provincial government, the BC-CfE launched the Seek and Treat for Optimal Prevention (STOP) HIV/AIDS initiative, a province-wide TasP program that included free ART for all PLWH regardless of disease stage, opt-out HIV testing for all BC residents, enhanced contact tracing, phylogenetic mapping of HIV clusters of transmission, and enhanced harm reduction [42,43]. Following implementation of STOP, a steady decline in new HIV diagnoses was observed (particularly among people who inject drugs), due to the combined effects of TasP and harm-reduction efforts [33]. However, the rate of decline was slower among gay, bisexual, and other men who have sex with men (gbMSM) [44]. Consequently, in January 2018, the BC-CfE added a fully subsidized PrEP program, primarily focused on gbMSM (at elevated risk of HIV infection), to further reduce HIV transmission in this population [44].

The ‘focused’ PrEP approach in BC has included offering PrEP, at no cost, to gbMSM, or transwomen with an elevated HIV Incidence Risk Index commonly referred to as the HI-RI MSM Score [45]. Since 2018, the BC PrEP program has provided free PrEP to 11,500 eligible individuals. Of note, the results presented here were obtained before long-acting antiretrovirals (i.e., lenacapavir) became available.

## 5. The Basic Reproduction Number ($R_{0}$)

The basic reproduction number ($R_{0}$) is a key epidemiological indicator that reflects the transmissibility of an infectious pathogen during a person’s lifetime [46,47,48,49,50]. For HIV, the $R_{0}$ represents the number of secondary infections generated from one primary infection over the infected person’s lifetime in a fully susceptible population. In contrast, the closely related effective reproduction number, ($R_{e}$), represents the average number of secondary infections from people living with HIV (PLWH) in a population where a proportion of individuals are already infected [51,52]. Both $R_{0}$ and $R_{e}$ are determined by three main parameters: the number of HIV-seronegative partners that a PLWH has in a given time period, the probability of transmitting HIV to a partner during that time, and the duration of infectiousness [45,46,47,48,49,50,51,52]. All three parameters can be affected by multiple factors, including TasP, PrEP, other preventive interventions, behavioural changes, and social or structural barriers [53,54]. When the reproduction number drops below one—indicating that each PLWH infects fewer than one person over their lifetime—the epidemic is declining. If this trend continues, the epidemic is expected to eventually end [45,46,47,48,49,50,51,52,53,54].

### Characterizing the Impact of TasP and PrEP on $R_{e}$

Recently, we conducted a longitudinal analysis to assess the impact of TasP and PrEP on R_e_ at the population level in BC [55]. Our study showed that the number of people on ART increased markedly in BC after 1992, with an overall increase of 256% since 1996 (*p* < 0.0001), while new HIV diagnoses went from its peak of 929 in 1987 to 134 in 2021 (a decrease of 86% (*p* < 0.0001)). Since 1985, there has been a steady decline in HIV infections incidence, from 818 cases in 1985 to 71 cases in 2022 (a decrease of 91% (*p* < 0.0001)). In the same period, the prevalence of PLWH rose from 3773 cases in 1985 to 9459 cases in 2022 (an increase of 151% (*p* < 0.0001)). Between 1993 and 2022, there were 4944 all-cause deaths of PLWH, of which 2311 (47%) were HIV/AIDS-related. We also observed that the trend in the HIV/AIDS-related mortality rates decreased from 1993 to 2022, with 221 individuals dying of HIV/AIDS in 1994 (peak) and only 15 individuals dying of HIV/AIDS in 2022. Using the overall BC population as a denominator, the HIV/AIDS-related mortality rate decreased from 6.0 to 0.3 per 100,000 population from 1994 to 2022 (a 95% decrease (*p* < 0.0001)). Of note, our results showed that for each increase of 100 individuals actively on ART, the estimated HIV incidence rate decreased by 2.5%, and for each increase of 100 individuals on PrEP, the estimated incidence rate fell by 1.7%.

**Figure 1:**
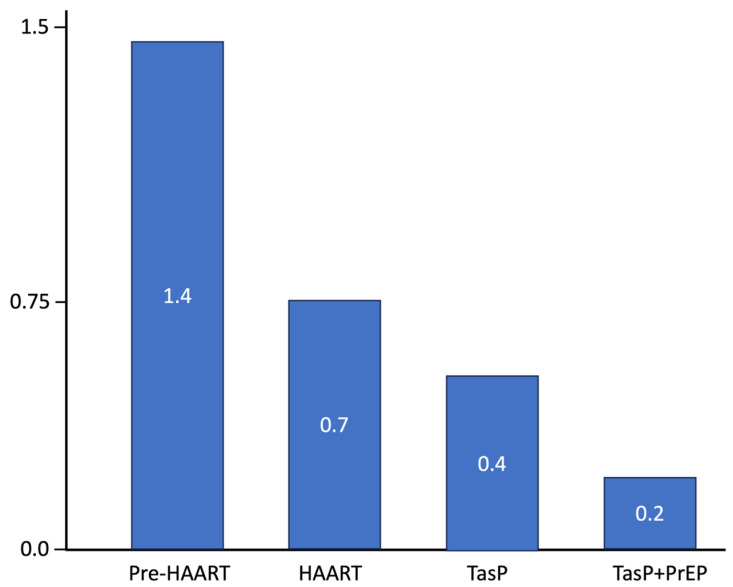
Estimated HIV effective reproduction number ($R_{e})\;$ over time in BC (the vertical axis refers to the “Effective Reproductive Number (Re)”and the horizontal axis shows the different eras of TasP and PrEP roll-out in BC, as described in the text).

As shown in Figure 1, the estimated $R_{e}$ for HIV has progressively declined across different phases in BC—from the pre-HAART era, to the initial roll-out of HAART, to the TasP era, and most recently, to the TasP + PrEP era. This figure visually supports the progressive reduction in HIV transmission over time, corresponding with the introduction of increasingly effective biomedical interventions, from HAART to TasP and PrEP. These data suggest that each intervention, particularly when combined (TasP + PrEP), contributes significantly to lowering the average number of secondary infections per PLWH, bringing $R_{e}$ closer to or below 1, which is crucial for epidemic control.

These results demonstrate the population-level effectiveness of TasP and PrEP significantly decreased HIV/AIDS-related morbidity, mortality, and HIV transmission from 1996 to 2022, when both strategies were jointly deployed in BC’s fully subsidized healthcare system. In 2022, the Public Health Agency of Canada estimated that, by the end of 2020, BC had already surpassed the UN 90-90-90 target and was nearing the UN 95-95-95 by 2025 target [55,56,57]. By 2020, 94% of PLWH in BC were diagnosed, 92% of those diagnosed were on ART, and 95% of individuals on ART were virologically suppressed. This translates to 94% of all PLWH being diagnosed, 86% receiving ART, and 82% being virologically suppressed [55,56,57].

Interestingly, our results revealed that HIV prevalence in BC increased until 2015, likely due to the improved survival experienced by PLWH on ART, which outweighed the significant reductions in HIV incidence. However, more recently, HIV prevalence in BC started to decline, potentially driven by rising age-related morbidity and mortality within the PLWH cohort. This demographic shift is evident in the median age of PLWH in BC, which steadily rose from 38 years (25th–75th percentile: 33–44 years) in 1996 to 55 years (25th–75th percentile: 45–62 years) by 2022 [43]. More recently, this has been compounded by the ubiquitous toxic drug poisoning crisis, which has been particularly severe in BC, especially among PLWH, who experienced an estimated 3.3 years’ loss of life expectancy due to overdose deaths [58].

**Figure 2:**
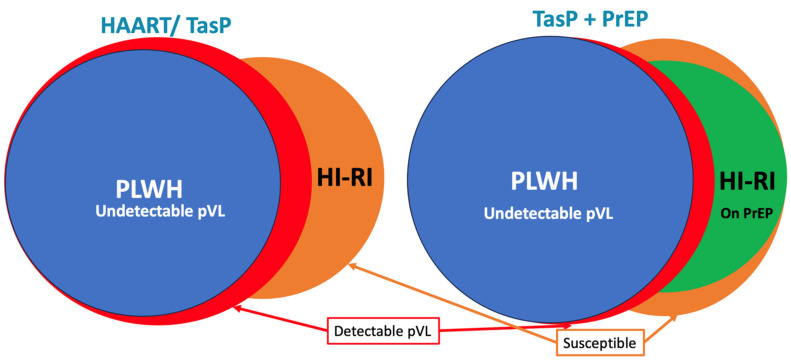
Schematic representation of the impact of HAART/TasP and TasP + PrEP on $R_{e}$. HAART: highly active antiretroviral therapy; TasP: Treatment as Prevention; PrEP: Pre-Exposure Prophylaxis; PLWH: people living with HIV; HI-RI: HIV negative gbMSM or transwomen with Elevated High-Risk (HIRI) score; pVL: plasma viral load.

For the first time, our results illustrate substantial longitudinal changes in R_e_ following the roll-out of antiretroviral-based initiatives at the population level. We observed an overall progressive and steady decline in $R_{e}$ among PLWH during the roll-out of HAART and TasP. However, $R_{e}$ remained unchanged for individuals with virologically unsuppressed HIV, regardless of whether the viral load threshold considered was 200 or 1000 copies/mL. This underscores that TasP reduces HIV incidence by lowering the number of people capable of transmitting the virus. Interestingly, when focused PrEP was introduced with a background of continued generalized TasP, $R_{e}$ continued to decline, both among the entire population of PLWH and when the analysis was restricted to individuals with detectable viral loads. The latter is consistent with the notion that PrEP decreases HIV incidence by decreasing the pool of susceptible individuals in the community, independent of the pVL among PLWH, schematically illustrated in Figure 2.

Of note, we estimated that for every increase of 100 individuals actively on ART, the HIV incidence rate decreased by 2.5%. Additionally, for every increase of 100 individuals on PrEP, against a background of continued TasP, the estimated incidence rate dropped by 1.7%. Importantly, these estimates focus solely on the impact of TasP and PrEP on HIV transmission, a common effect of both interventions. However, these estimates do not account for the well-documented and profound impact of ART on reducing HIV-related morbidity and mortality, which is specific to ART use among PLWH.

While the specific number or proportion of individuals that need to be on ART or PrEP to optimize the populational impact of the strategy will vary according to the various characteristics of the epidemic in each jurisdiction (socio-demographics, epidemiology, access to care, etc.), it is reassuring that studies derived from populations across the world have consistently shown this approach is not only effective, but also economically sound. For example, ART-based Treatment as Prevention has been shown to be consistently cost-saving in a BC-based model by Johnston et al. [59], a global model by Granich et al. [60], a South Africa model by Walensky et al. [61], and in North America by Nosyk et al. [62] With regard to PrEP, Gomez et al., in a systematic review of modelling studies [63], concluded that PrEP can be cost-effective in some settings, but this is dependent on factors such as drug cost, epidemic context, coverage, prioritization strategies, adherence levels, and efficacy. These findings are supported by the secondary analyses from the iPrEx trial, as reported by Buchbinder et al. [26,64], concluding that focusing on high-risk individuals is critical to the cost-effectiveness of PrEP.

## 6. TasP + PrEP in Other Jurisdictions

The combination of TasP + PrEP has been evaluated in various settings globally, with mixed results. To illustrate this, it suffices to look at the Canadian experience. In brief, Canada endorsed the 90-90-90 by 2020 and subsequent 95–95–95 by 2025 targets. Yet, a total of 2434 new HIV cases were diagnosed in Canada in 2023, which represents a 35% increase since 2022. Therefore, the overall rate of new diagnoses per 100,000 people in Canada increased from 4.7 to 6.1 between 2022 and 2023. Outside of the smaller jurisdictions (i.e., territories and Atlantic Canada), BC had the lowest rate of HIV new diagnoses at 3.3/100,000 people. Of note, BC had the highest rates of HIV new infections in the early phase of the epidemic. Currently, the remainder of the HIV diagnosis rates in Canada are as follows: Territories: 2.2/100,000 people, British Columbia: 3.3/100,000 people, Alberta: 5.4/100,000 people, Saskatchewan: 19.4/100,000 people, Manitoba: 19.3/100,000 people, Ontario: 6.0/100,000 people, Quebec: 5.4/100,000 people, Atlantic: 2.4/100,000 people [65].

While the reasons for the disparities between Canadian jurisdictions are multifactorial, it is clear that political will, public health leadership, and community involvement are key to the success of the strategy. Similar disparities are apparent elsewhere globally, as illustrated in the most recent UNAIDS report [66]. Within this context, it is reassuring that in Greece, a similar program was successfully deployed at a time when the HIV epidemic was deemed to be out of control (particularly among people who use injection drugs), largely due to the devastating consequences of the economic crisis. In Athens, the initiative provided supported HIV testing, counselling, and linkage to care among people who use injection drugs. As a result, the 2011 HIV outbreak was successfully contained [67,68].

## 7. Conclusions

In summary, our population-level experience in BC within a fully subsidized healthcare environment demonstrates the unparalleled ability of generalized TasP, combined with focused PrEP, in substantially reducing HIV/AIDS-related morbidity, mortality, and HIV incidence. Our comprehensive analysis of longitudinal changes in the effective reproduction number (R_e_) reveals its steady and progressive decline over nearly three decades. While the effect of generalized TasP on HIV incidence was driven by its ability to decrease the pool of infectious PLWH, the addition of focused PrEP further decreased HIV incidence by reducing the number of susceptible individuals in the community. Notably, the impact of PrEP was independent of viral suppression among PLWH. These results provide strong support for widespread implementation of the “generalized TasP + focused PrEP” strategy and highlight the importance of ongoing longitudinal monitoring of R_e_ at the programmatic level. As we move forward, this approach will be critical in achieving the United Nations’ goal of “Ending HIV/AIDS as a pandemic by 2030” in Canada and globally.

## Data Availability

Data sharing is not applicable.
